# Respective impact of implementation of prevention strategies, colonization with multiresistant bacteria and antimicrobial use on the risk of early- and late-onset VAP: An analysis of the OUTCOMEREA network

**DOI:** 10.1371/journal.pone.0187791

**Published:** 2017-11-29

**Authors:** Wafa Ibn Saied, Bertrand Souweine, Maité Garrouste-Orgeas, Stéphane Ruckly, Michael Darmon, Sébastien Bailly, Yves Cohen, Elie Azoulay, Carole Schwebel, Aguila Radjou, Hatem Kallel, Christophe Adrie, Anne-Sylvie Dumenil, Laurent Argaud, Guillaume Marcotte, Samir Jamali, Laurent Papazian, Dany Goldgran-Toledano, Lila Bouadma, Jean-Francois Timsit, for the OUTCOMEREA study group

**Affiliations:** 1 UMR 1137 - IAME Team 5 – DeSCID: Decision SCiences in Infectious Diseases, control and care, Inserm/ Paris Diderot University, Sorbonne Paris Cité, Paris, France; 2 Medical Intensive care unit, Grenoble University Hospital, Grenoble 1 University, U823, La Tronche, France; 3 Medical Intensive Care Unit, Gabriel Montpied University Hospital, Clermont-Ferrand, France; 4 Intensive Care Unit, Saint Joseph Hospital Network, Paris, France; 5 Saint Etienne University Hospital, Medical Intensive Care Unit, Saint-Etienne, France; 6 Grenoble Alpes University, U823, Rond-point de la Chantourne, La Tronche France; 7 AP-HP, Avicenne Hospital, Intensive Care Unit, Paris and Medicine University, Paris 13 University, Bobigny, France; 8 Medical Intensive Care Unit, AP-HP, Saint Louis Hospital, Paris, France; 9 AP-HP, Bichat Hospital, Medical and infectious diseases Intensive Care Unit, Paris Diderot university, Paris, France; 10 Medical Surgical ICU, Centre hospitalier de Cayenne, Guyane, France; 11 Physiology department, Cochin University Hospital, Sorbonne Cite, Paris, France; 12 AP-HP, Antoine Béclère University Hospital, Medical-surgical Intensive Care Unit, Clamart, France; 13 Medical Intensive Care Unit, Lyon University Hospital, Lyon, France; 14 Surgical ICU, Edouard Herriot University Hospital, Lyon, France; 15 Critical care Medicine Unit Dourdan Hospital, Dourdan, France; 16 Respiratory and infectious diseases ICU, APHM Hôpital Nord, Aix Marseille University, Marseille, France; 17 Gonesse Hospital, Intensive Care Unit, Gonesse, France; National Yang-Ming University, TAIWAN

## Abstract

**Rationale:**

The impact of prevention strategies and risk factors for early-onset (EOP) versus late-onset (LOP) ventilator-associated pneumonia (VAP) are still debated.

**Objectives:**

To evaluate, in a multicenter cohort, the risk factors for EOP and LOP, as the evolution of prevention strategies.

**Methods:**

7,784 patients with mechanical ventilation (MV) for at least 48 hours were selected into the multicenter prospective OUTCOMEREA database (1997–2016). VAP occurring between the 3^rd^ and 6^th^ day of MV defined EOP, while those occurring after defined LOPs. We used a Fine and Gray subdistribution model to take the successful extubation into account as a competing event.

**Measurements and main results:**

Overall, 1,234 included patients developed VAP (EOP: 445 (36%); LOP: 789 (64%)). Male gender was a risk factor for both EOP and LOP. Factors specifically associated with EOP were admission for respiratory distress, previous colonization with multidrug-resistant *Pseudomonas aeruginosa*, chest tube and enteral feeding within the first 2 days of MV. Antimicrobials administrated within the first 2 days of MV were all protective of EOP. ICU admission for COPD exacerbation or pneumonia were early risk factors for LOP, while imidazole and vancomycin use within the first 2 days of MV were protective factors. Late risk factors (between the 3^rd^ and the 6^th^ day of MV) were the intra-hospital transport, PAO2-FIO2<200 mmHg, vasopressor use, and known colonization with methicillin-resistant *Staphylococcus aureus*. Among the antimicrobials administered between the 3^rd^ and the 6^th^ day, fluoroquinolones were the solely protective one.Contrarily to LOP, the risk of EOP decreased across the study time periods, concomitantly with an increase in the compliance with bundle of prevention measures.

**Conclusion:**

VAP risk factors are mostly different according to the pneumonia time of onset, which should lead to differentiated prevention strategies.

## Introduction

Ventilator-acquired pneumonia (VAP) is defined as pneumonia arising after at least 48 hours of mechanical ventilation (MV). VAP represents the leading type of nosocomial infection in intensive care units (ICUs); its rate ranges from 8 to 25% among mechanically ventilated patients [1]. VAP is associated with a significant increase in the mortality risk for ICU patients, from 3 to 8% when using new statistical model for causal inference [2, 3]. It increases the length of ICU stay by up to 9 days [1].

The major route for acquiring VAP is oropharyngeal colonization by the endogenous flora or by pathogens acquired exogenously from the intensive care unit environment, especially the hands of health-care workers or the contaminated respiratory equipment. The stomach represents a potential site of secondary colonization and reservoir of nosocomial Gram-negative bacilli. On endotracheal-tube, the formation of biofilm may play a contributory role in sustaining tracheal colonization, paving the way for VAP mostly caused by resistant organisms such as methicillin-resistant *staphylococcus aureus* (MRSA) and non-fermentative gram-negative bacilli (GNB). The immunoparalysis occurring after the initial phase of resuscitation may also play a role in decreasing resistance to infections [4–6]

The changes in the pathogenesis of VAP over time have led to differentiate early-onset pneumonia (EOP) from late-onset pneumonia (LOP) [7]. Time-points used for differentiating them are slightly different according to studies, from >3 to ≥7 days of mechanical ventilation [8–10].

According to the differences in the pathogenesis, different preventive measures might be implemented. Indeed, the rapid decrease in the endogenous flora inoculum at the early phase of ventilation by antimicrobial prophylaxis [11], selective digestive and oral decontamination, subglottic aspiration [12, 13], semi-recumbent position [14], decreased the risk of EOP, but have no or a moderate impact on LOP. When preventive bundles such as weaning trials, orogastric tubes, semi-recumbent position, or oral care were applied, the decrease in VAP rates was greatest for the period within 5 days after ICU admission and MV initiation, suggesting that preventive measures were more effective when implemented early[6]. On the opposite, it was shown that the constitution of a secondary reservoir in the stomach promotes infections occurring later that might be partly prevented by the preservation of a low gastric pH [15].

The aim of our study was to assess specific risk factors for EOP and for LOP in a large multicenter database, and the impact of the progressive implementation of recommended preventive strategies on the rates of onset of EOP and of LOP.

## Methods

### Study design and data source

**Fig 1:**
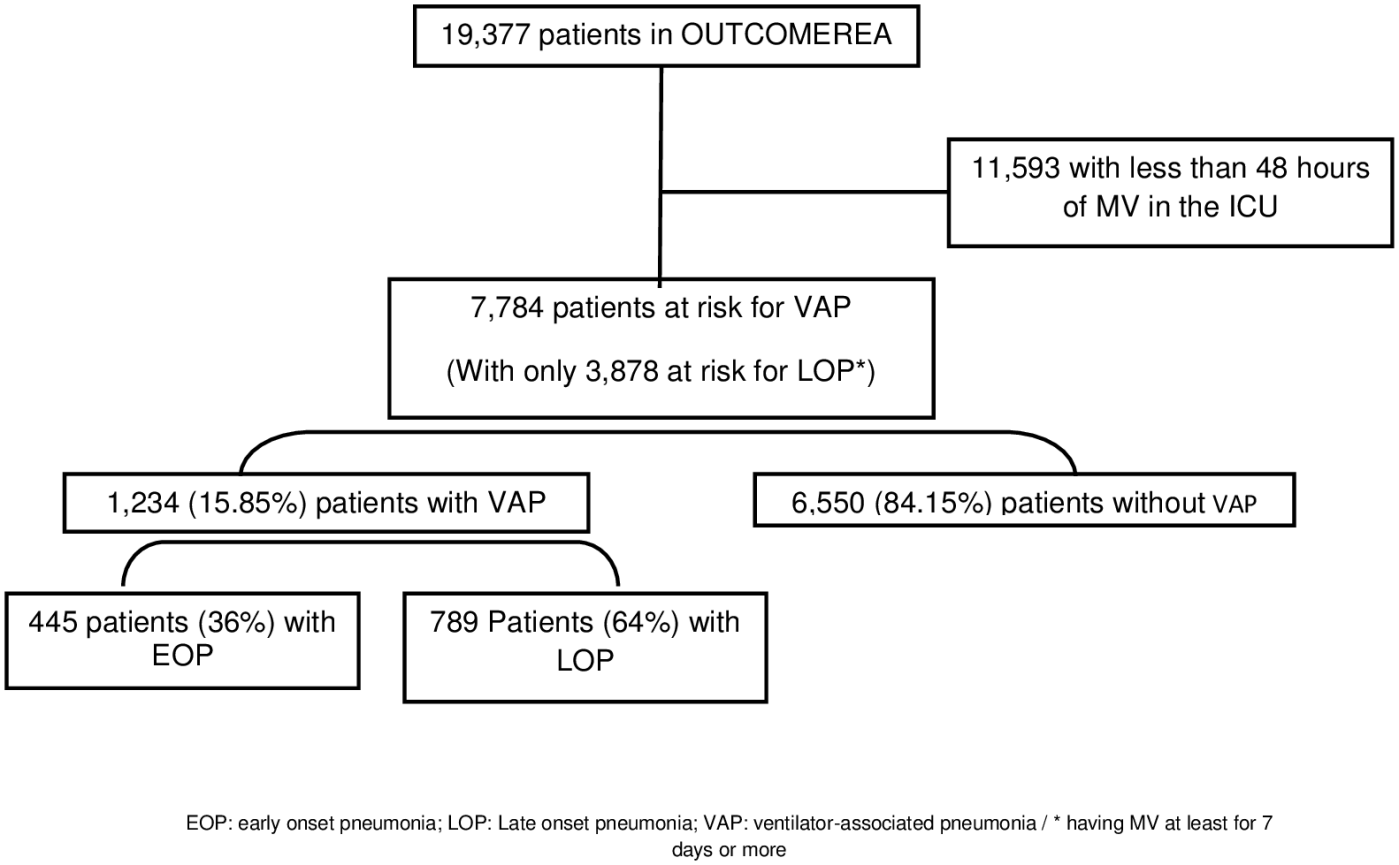
Flowchart.

We conducted a retrospective analysis using data prospectively entered into a multicenter database from January 1997 to March 2016 (OUTCOMEREA database). The database is fed by 22 different French ICUs, and contains data on admission features and diagnosis, daily disease severity, iatrogenic events, nosocomial infections, and vital status. ICU units participating in the OUTCOMEREA database used a range of methods to include patients. Some ICUs included all consecutive patients, while other units included patients admitted at predefined periods of the year, and some other picked patients in certain beds that were randomly selected, without considering patient's characteristics such as the diseases at ICU admission or their medical history [16]. All patients with MV lasting more than 48 hours were included into the study. Patients were censored after 28 days of MV or death (Fig 1). Risk factors were evaluated for the first episode of EOP on the individuals under mechanical ventilation for more than 2 days, and a similar analysis was performed for the first episode of LOP on the patients who were still mechanically ventilated on the 7^th^ day.

#### Ethical issues

This study was approved by our institutional review board (CECIC Clermont-Ferrand—IRB n°5891; Ref: 2007–16), which waived the need for signed informed consent of the participants, in accordance with French legislation on non-interventional studies. However, the patients and their next of kin were asked whether they were willing to participate in the database, and none declined participation.

#### Population definitions

The diagnosis of VAP was suspected in patients who had received at least 48 hours of MV and developed a new or persistent infiltrate on chest radiography, associated with one of the following criteria: (1) purulent tracheal secretions, (2) fever greater than or equal to 38.5°C or hypothermia less than or equal to 36.5°C, and (3) leukocytosis greater than 10^9^ cells/L or leukopenia less than 4.10^8^ cells/L. The diagnosis of VAP was confirmed by a positive quantitative culture of a respiratory sample: bronchoalveolar lavage fluid (significant threshold: ≥ 10^4^cfu/ml) or plugged telescopic catheter (significant threshold: ≥ 10^3^cfu/ml) or quantitative endotracheal aspirate (significant threshold, ≥10^5^ cfu/ml). The model was only based on confirmed VAPs with a positive result after bacteriological culture. When VAP was suspected, samples were performed prior to any changes in the antibiotic therapy.

We chose a cut-off point at day 7 of MV. EOP was defined as the occurrence of VAP between the 3^rd^ and the 6^th^ day of MV. LOP was defined as the occurrence of VAP after the 6^th^ day of MV.

A new episode of VAP was defined either by: (1) the same causative microorganism after a time interval of at least 5 days from the previous episode; (2) the acquisition of resistance of the same pathogen; or (3) a different pathogen regardless the acquisition day. Positive samples for fungi or viruses were not considered.

We also recorded data on previous carriage or colonization with MDR bacteria: multi-drug-resistant *P*. *aeruginosa* (MDRPA), MRSA, extended*-*spectrum β*-*lactamase producing *Enterobacteriaceae* (ESBLE), and *A*. *baumannii*. A patient was considered as colonized with MDR bacteria if one of these 4 microorganisms was isolated from clinically-indicated samples, or from samples drawn for systematic detection for MRSA and ESBLE, i.e., samples collected at admission and weekly, from nasal and rectal swabs for MRSA and ESBLE, respectively. If confirmed, patient was considered as carrier throughout the full study period.

#### Data collection

The following data were collected at admission, and daily if appropriate: age; sex; comorbidities (assessed according to the Acute Physiology and Chronic Health Evaluation II definitions); severity of illness at ICU admission and daily during the ICU stay (assessed using the Simplified Acute Physiology Score II and the Logistic Organ Dysfunction score); admission category (medical, surgery, or monitoring); admission diagnosis; whether the patient was transferred from a hospital ward (defined as a stay in an acute-bed ward lasting>24 h immediately before ICU admission); lengths of ICU stay and hospital stay; and vital status at discharge from the ICU and from the hospital. Data on invasive procedures (placement of an arterial or central venous catheter, and endotracheal intubation), treatments of organ failures (catecholamine infusion, mechanical ventilation, renal replacement therapy), colonization with ESBLE, MDRPA and MRSA, and antibiotic use were also captured daily.

Prevention bundle applied during each of the study periods: before 2001, between 2001 and 2006 and after 2006, was recorded using questionnaires filled by investigators (see description in S1 Table). The compliance with prevention protocols was not monitored. We described EOP and LOP tendency taking account of the modification of bundles over these 3 periods.

#### Statistical analysis

Characteristics of patients and VAP episodes were described using frequency and percentage for qualitative variables, median, and interquartile range (IQR) for quantitative variables. Standard survival analyses are affected by the time of onset of the event of interest. Patients who have not experienced the event at the end of follow-up were censored. To determine the risk of an event occurrence at a certain time-point, a fundamental assumption is that such censoring is not associated with an altered chance of the event occurring at any given moment. In this study, the event of interest is the onset of pneumonia in patients with MV and followed up until day 28 or until extubation for more than 48 hours. Indeed, death and extubation for more than 48 hours is a competing event since, by definition, extubation precludes the observation of a ventilator-associated infection [17]. Risk factors of VAP (EOP and LOP) occurrence were identified separately using Fine and Gray models stratified by ICU. A multivariate model was built by including risk factors (at admission, the 2 first days of MV for EOP, and between the third and the sixth day of MV for LOP) that met the 20% significance threshold in univariate analysis. SAPS II at the beginning of mechanical ventilation period was selected for the model, instead of organ failure scores and age. Then, a backward selection process was applied until all remaining factors met the 5% threshold in the multivariate context. For EOP as for LOP, some factors are specific to the patient, such as the male gender; others were recorded at ICU admission, and others emerged later on, up to the 3^rd^ to the 6^th^ day after MV initiation for LOP. Sensitivity Analysis was also performed by varying MV cut-off to five days for the definition of EOP and LOP without modification in the final model (S2 Table). All models were stratified by centers and adjusted on time periods and SAPS II at the beginning of mechanical ventilation period that correspond to the main changes in prevention strategies. Results were given as subdistribution hazard ratios (sHR) and 95% confidence intervals (CI). Using P values less than 0.05 were considered as significant. As it is an exploratory trial, p-values were further corrected for multiple comparisons using the Benjamini-Hochberg procedure, as implemented in the MULTITEST procedure in SAS. Statistical analysis was performed using SAS 9.4 (Cary, NC).

## Results

Overall, 7,784 ICU patients with mechanically ventilation for more than 48 hours were included *(see CONSORT flowchart*, Fig 1*)*, including 1,234 (15.85%) patients who developed VAP within the first 28 days of MV. Among them, 445 (36%) developed an EOP, and 789 (64%) developed a LOP. Characteristics at ICU admission, during the first 48 hours of MV and between the third day of MV and the sixth day of MV for patients at risk for EOP and LOP are described in Tables 1 and 2 respectively. The main causative pathogens according to the type of VAP are described in Table 3.

**Table 1:** Characteristics of study population at the ICU admission.

Variables at ICU admission	Patients at risk for EOP (N = 7,784)			Patients at risk for LOP (N = 3,878)		
	No EOP (N = 7,339)	EOP (N = 445)	P*	No LOP (N = 3,089)	LOP (N = 789)	P**
**Age (#miss = 5)**	64.6 [52.1; 75.8]	64.1 [51.2; 72.8]	0.09	66.6 [54.6; 76.3]	65.1 [53.2; 75.7]	0.02
**Male gender**	4,564 (62.2)	312 (70.1)	<.01	1,939 (62.8)	545 (69.1)	<.01
**Chronic diseases at ICU admission**						
Hepatic	528 (7.2)	22 (4.9)	0.07	197 (6.4)	57 (7.2)	0.39
Cardiovascular	1,049 (14.3)	67 (15.1)	0.66	491 (15.9)	110 (13.9)	0.18
Respiratory	1,182 (16.1)	78 (17.5)	0.43	587 (19)	165 (20.9)	0.23
Renal	355 (4.8)	30 (6.7)	0.07	147 (4.8)	29 (3.7)	0.19
Immunodeficiency	1,184 (16.1)	54 (12.1)	0.03	519 (16.8)	145 (18.4)	0.29
**Diabetes**						0.32
Complicated diabetes	333 (4.5)	19 (4.3)	0.71	144 (4.7)	39 (4.9)	
Non complicated diabetes	810 (11)	44 (9.9)		351 (11.4)	75 (9.5)	
No diabetes	6,196 (84.4)	382 (85.8)		2,594 (84)	675 (85.6)	
**Diagnosis category at ICU admission (#miss = 18)**						0.03
Scheduled surgery	639 (8.7)	40 (9)	0.05	242 (7.8)	65 (8.2)	
Emergency surgery	1,383 (18.8)	63 (14.2)		581 (18.8)	117 (14.8)	
Medical	5,317 (72.4)	342 (76.9)		2,266 (73.4)	607 (76.9)	
**Diagnosis at ICU admission**						
Septic shock	1,486 (20.2)	46 (10.3)	<.01	777 (25.2)	169 (21.4)	0.03
Other shock	1,088 (14.8)	69 (15.5)	0.70	444 (14.4)	15 (1.9)	0.56
Respiratory distress	2,172 (29.6)	157 (35.3)	0.01	1,090 (35.3)	252 (31.9)	<.01
COPD exacerbation	412 (5.6)	26 (5.8)	0.84	213 (6.9)	73 (9.3)	0.05
Coma	1,759 (24)	131 (29.4)	<.01	530 (17.2)	127 (16.1)	0.48
Pneumonia	1,507 (20.5)	105 (23.6)	0.12	773 (25)	252 (31.9)	<.01
Drug overdose	384 (5.2)	23 (5.2)	0.95	52 (1.7)	13 (1.6)	0.94

P* = p value comparing EOP and No EOP patients /P** = p value comparing LOP and No LOP patients; EOP early onset pneumonia; LOP: late onset pneumonia; MV: mechanical ventilation; COPD exacerbation = chronic obstructive pulmonary disease exacerbation; MV = mechanical ventilation; # miss: number of missing data

**Table 2:** Characteristics of patients at risk for early and late onset pneumonia during their stay in ICU.

Characteristics	Patients at risk for EOP (N = 7,784)			Patients at risk for LOP (N = 3,878)		
	No EOP (N = 7,339)	EOP (N = 445)	P*	No LOP (N = 3,089)	LOP (N = 789)	P**
**Variables within the first 48 hours of MV**						
**SAPSII score**	48 [37; 59]	47 [37; 58]		50 [40; 60]	48 [39; 59]	
≤38	2,034 (27.7)	121 (27.2)	0.33	701 (22.7)	196 (24.8)	0.18
37–48	1,762 (24)	121 (27.2)		741 (24)	207 (26.2)	
49–59	1,716 (23.4)	106 (23.8)		837 (27.1)	193 (24.5)	
≥60	1,827 (24.9)	97 (21.8)		810 (26.2)	193 (24.5)	
**Effective Glasgow**†	4 [3; 8]	4 [3; 7]		4 [3; 8]	3 [3; 7]	
≤ 5	3,693 (50.3)	237 (53.3)	0.02	1,559 (50.5)	453 (57.4)	<.01
5>Glasgow<13	2,761 (37.6)	174 (39.1)		1,194 (38.7)	255 (32.3)	
≥ 13	885 (12.1)	34 (7.6)		336 (10.9)	81 (10.3)	
**PAO_2:_FIO2, median (IQR) (# miss = 150)**						
<200mm Hg	3783 (51.5)	233 (52.4)	0.74	1766 (57.2)	515 (65.3)	<.01
≥200mm Hg	3556 (48.5)	212 (47.6)		1,364 (44.2)	274 (34.7)	
**Microbial colonization**						
ESBLE	186 (2.5)	11 (2.5)	0.94	98 (3.2)	22 (2.8)	0.58
MDR PA	76 (1)	9 (2)	0.05	38 (1.2)	12 (1.5)	0.52
*Acinetobacter baumannii*	216 (2.9)	12 (2.7)	0.76	113 (3.7)	27 (3.4)	0.75
MRSA	279 (3.8)	18 (4)	0.79	141 (4.6)	38 (4.8)	0.76
**Treatments and procedures**						
Unplanned extubation	200 (2.7)	13 (2.9)	0.81	59 (1.9)	19 (2.4)	0.37
Chest tube	566 (7.7)	54 (12.1)	<.01	263 (8.5)	84 (10.6)	0.06
Steroids	2,049 (27.9)	100 (22.5)	0.01	982 (31.8)	262 (33.2)	0.45
Paralytic agents	1,477 (20.1)	98 (22)	0.33	699 (22.6)	230 (29.2)	0.16
Proton Pump Inhibitors	4,526 (61.7)	250 (56.2)	0.02	1,940 (62.8)	471 (59.7)	0.11
Enteral feeding	2,320 (31.6)	177 (39.8)	<.01	1,122 (36.3)	307 (38.9)	0.18
Intra-hospital transport	2,069 (28.2)	108 (24.3)	0.07	856 (27.7)	220 (27.9)	0.92
Patient isolation	1,288 (17.6)	64 (14.4)	0.09	574 (18.6)	151 (19.1)	0.72
**Antibacterial agents administration**						
3^rd^ and 4^th^ generation cephalosporins	1,810 (24.7)	76 (17.1)	<.01	901 (29.2)	246 (31.2)	0.33
ß-lactam/ ß-lactamase inhibitor	2,691 (36.7)	135 (30.3)	<.01	1,107 (35.8)	295 (37.4)	0.09
Other penicillins	755 (10.3)	34 (7.6)	0.07	353 (11.4)	86 (10.9)	0.68
Penems	600 (8.2)	10 (2.2)	<.01	323 (10.5)	64 (8.1)	0.05
Fluoroquinolones	1,205 (16.4)	44 (9.9)	<.01	633 (20.5)	151 (19.1)	0.40
Aminoglycosides	1,784 (24.3)	51 (11.5)	<.01	914 (29.6)	202 (25.6)	0.03
Imidazole	849 (11.6)	30 (6.7)	<.01	448 (14.5)	86 (10.9)	0.71
Vancomycin	956 (13)	27 (6.1)	<.01	519 (16.8)	94 (11.9)	<.01
Other antibacterial agents	825 (11.2)	57 (12.8)	0.31	322 (10.4)	100 (12.7)	0.07
**Variables between the 3 ^rd^ and the 6^th^ days of MV**						
**SAPSII score**	-	-		46 [37; 56]	46 [39; 57]	0.51
≤37				713 (23.1)	162 (20.5)	
37–57				1,660 (53.7)	442 (56)	
≥57				716 (23.2)	185 (23.4)	
**Effective Glasgow**†	-	-		6 [3; 10]	5 [3; 8]	<.01
≤ 5				1,192 (38.6)	379 (48)	
5>Glasgow<13				1484 (48)	319 (40.4)	
≥ 13				413 (13.4)	91 (11.5)	
**PAO_2:_FIO2, median (IQR)**	-	-				
<200mm Hg				1,499 (48.5)	492 (62.4)	<.01
≥200mm Hg				1,590 (51.5)	297 (37.6)	
**Microbial colonization**	-	-				
ESBLE				119 (3.9)	31 (3.9)	0.92
MDR PA				34 (1.1)	16 (2)	0.04
*Acinetobacter baumannii*				142 (4.6)	37 (4.7)	0.91
MRSA				123 (4)	44 (5.6)	0.05
**Treatments and procedures**	-	-				
Unplanned extubation				140 (4.5)	40 (5.1)	0.52
Chest tube				311 (10.1)	107 (13.6)	<.01
Steroids				1,132 (36.6)	309 (39.2)	0.19
Paralytic agents				429 (13.9)	179 (22.7)	<.01
Proton Pump Inhibitors				2,121 (68.7)	531 (67.3)	0.46
Enteral feeding				921 (29.8)	202 (25.6)	0.02
Intra-hospital transport				610 (19.7)	189 (24)	<.01
Patient isolation				610 (19.7)	166 (21)	0.42
**Antibacterial agents administration**	-	-				
3^rd^ and 4^th^ generation cephalosporins				989 (32)	265 (33.6)	0.40
ß-lactam/ ß-lactamase inhibitor				1,149 (37.2)	303 (38.4)	0.53
Other penicillins				533 (17.3)	146 (18.5)	0.41
Penems				411 (13.3)	85 (10.8)	0.06
Fluoroquinolones				641 (20.8)	140 (17.7)	0.06
Aminoglycosides				805 (26.1)	193 (24.5)	0.36
Imidazole				542 (17.5)	110 (13.9)	0.02
Vancomycin				549 (17.8)	127 (16.1)	0.27
Other antibacterial agents				559 (18.1)	182 (23.1)	<.01
**Early-onset pneumonia**				85 (9.7)	290 (8.8)	0.29

P** = p value comparing LOP and No LOP patients; ICU: intensive care unit; EOP early onset pneumonia; LOP: late onset pneumonia; MV: mechanical ventilation; SAPSII score = Simplified Acute Physiology Score within the first 48h after ICU admission;

^†^ GLASGOW score = Glasgow coma scale within the first 48h after ICU admission—scored even in patients receiving sedation—represents the level of awakening of patients; MDRPA = multi-drug-resistant *Pseudomonas aeruginosa*; MRSA = Methicillin-resistant *Staphylococcus aureus*; ESBL-PE: extended-spectrum βlactamase producing *Enterobacteriaceae*; # miss: number of missing data

**Table 3:** Causative pathogens according to the type of ventilator-associated pneumonia, EOP or LOP.

Pathogens	EOP (N = 445)	LOP (N = 789)	pvalue
Staphylococcus aureus S*	100 (16.9)	141 (14.7)	0.24
Staphylococcus aureus MDR**	23 (5.1)	71 (8.8)	0.02
Staphylococcus coagulase negative /epidermis S*	18 (3.1)	48 (5)	0.06
Staphylococcus coagulase negative /epidermis MDR**	6 (1.3)	27 (3.4)	0.03
***Acinetobacter baumannii***	14 (2.4)	24 (2.5)	0.87
Pseudomonas aeruginosa S*	108 (18.3)	278 (29)	<.01
Pseudomonas aeruginosa MDR**	25 (5.6)	96 (11.9)	<.01
***Stenotrophomonas maltophilia***	14 (2.4)	42 (4.4)	0.04
**Other Non fermenting Gram negative Bacteria✉**	2 (0.3)	8 (0.8)	0.24
Escherichia coli S*	35 (5.9)	76 (7.9)	0.14
Escherichia coli MDR**	7 (1.6)	12 (1.5)	0.92
*Klebsiella pneumoniae /Proteus spp*. S*	47 (8)	70 (7.3)	0.63
*Klebsiella pneumoniae / Proteus spp*. MDR**	5 (1.1)	10 (1.2)	0.84
***Serratia/ Citrobacter freundii/ Enterobacter cloacae S*** *	78 (13.2)	114 (11.9)	0.44
Serratia/ Citrobacter freundii/ Enterobacter cloacae MDR**	13 (2.9)	40 (5)	0.08
***Haemophilus influenzae***	58 (9.8)	36 (3.8)	<.01
***Streptococcus pneumoniae***	35 (5.9)	31 (3.2)	0.01
**Other streptococci❖**	32 (5.4)	24 (2.5)	<.01
**Other pathogens ♦**	49 (8.3)	67 (7)	0.34

*S: susceptible strains,

**: MDR: multi-drug resistant strains

✉Other Non fermenting GNB: *Pseudomonas putida* and other *Pseudomonas* spp.;, *Acinetobacter baumannii* ❖Other streptococci: group A streptococci, group B streptococci—Beta-haemolytical streptococci, group C streptococci; **other pathogens** ♦: other gram positive cocci, *Neisseria meningitidis*, *Moraxella catarrhalis*, other *Bacillus* spp., *Corynebacterium* spp, *Lactobacillus*, *Klebsiella* spp., *Serratia* spp., other aerobic Gram negativebacilli, *Peptostreptoccus*, *Coxiella burnetti*, *Mycoplasma pneumoniae*, *Legionella* spp, *Aspergillus fumigatus*

Incidence of EOP, but not of LOP, decreased with the study time periods. Risk factors selected separately for EOP and LOP at the final step of the multivariate models are reported on Table 4. Male gender was the only risk factor common for EOP and LOP.

**Table 4:** Summary of risk factors of early- and late-onset pneumonia.

Variables	Early-Onset Pneumonia		Late-Onset Pneumonia	
	sHR	p	sHR	p
**Male gender**	1.33 [1.09–1.63]	<.01	1.23 [1.06–1.43]	0.0064
**Chronic diseases at ICU admission**				
Renal	1.49 [1.02–2.16]	0.04		
Immunodeficiency			1.27 [1.05–1.53]	0.012
Diagnosis at ICU admission				
Respiratory distress	1.28 [1.04–1.57]	0.02		
COPD exacerbation			1.24 [1.06–1.45]	0.0062
Pneumonia			1.26 [1.08–1.47]	<.01
**SAPSII score (ref: ≤38)**		0.25		0.0003
SAPSII ≥ 60	0.87 [0.65–1.16]	0.36	0.69 [0.57–0.84]	0.0003
48 ≤ SAPSII < 60	1.02 [0.78–1.33]	0.92	0.72 [0.59–0.88]	0.001
38 ≤ SAPSII < 48	1.15 [0.90–1.48]	0.26	0.92 [0.76–1.12]	0.393
**Glasgow score**†(ref:>13)		0.04		
5 ≤ Glasgow < 13	1.62 [1.10–2.36]	0.01		
Glasgow < 5	1.55 [1.06–2.27]	0.02		
**Period effect (ref: <2001)**		0.01		0.277
2001–2006	0.84 [0.58–1.21]	0.34	1.25 [0.95–1.64]	0.114
≥ 2007	0.63 [0.44–0.91]	0.01	1.22 [0.92–1.63]	0.161
**Variables within the first 48 hours of MV**				
Chest Tube	1.71 [1.30–2.26]	<.01		
MDR PA colonization	2.12 [1.08–4.16]	0.03		
Enteral feeding	1.34 [1.09–1.66]	<.01		
**Antibacterial agents**				
3 ^rd^ and 4 ^th^ generation cephalosporins	0.50 [0.38–0.65]	<.01		
ß lactam/ ß lactamase inhibitor	0.60 [0.48–0.75]	<.01		
Other penicillin	0.60 [0.42–0.87]	<.01		
Penems	0.27 [0.14–0.50]	<.01		
Aminoglycosides	0.57 [0.42–0.78]	<.01		
Fluoroquinolones	0.48 [0.35–0.68]	<.01		
Imidazoles			0.79 [0.63–0.99]	0.046
Vancomycin	0.62 [0.42–0.92]	0.02	0.65 [0.53–0.79]	<.0001
**Variables between the 3 ^rd^ and the 6^th^ days of MV**				
Fluoroquinolones			0.65 [0.53–0.79]	<.0001
Other Antibacterial agents			1.25 [1.05–1.49]	0.011
Intra-hospital transport			1.20 [1.01–1.42]	0.037
Colonization with MRSA			1.40 [1.01–1.93]	0.040
PAO_2_: FIO2 ratio <200 mmHg			1.49 [1.29–1.73]	<.0001

**Variables introduced in the model of risk factor of EOP at the first step of the selection procedure were**: age, male gender, chronic hepatic diseases, chronic renal diseases, immunodeficiency, diagnostic categories, septic shock. Variables within the first 48 hours of MV: chest tube, MDR PA colonization, steroids, Proton Pump Inhibitors, enteral feeding, intra-hospital transport, Patient isolation, Septic shock, Respiratory distress, coma, Aminoglycosides, 3^rd^ and 4^th^ generation cephalosporins, Penems, Fluoroquinolones, Vancomycin, Metronidazole, other penicillin, ß lactam/ ß lactamase inhibitor

**Variables introduced in the model of risk factor of LOP at the first step of the selection procedure were**: Septic shock, Respiratory distress, COPD exacerbation pneumonia, male gender, Variables within the first 48 hours of MV; chest tube, Proton Pump Inhibitors, SAPSII score Imidazole, other Antibacterial agent, Vancomycin, Penems, Aminoglycosides, Variables between the 3 ^rd^ and the 6 days of MV: PAO2FIO2, GLASGOW score, Metronidazole, other Antibacterial agent, Fluoroquinolones, Penems, intra-hospital transport, enteral feeding, vasopressor, MDR PA and MRSA colonization

ICU: intensive care unit; EOP early onset pneumonia; LOP: late onset pneumonia; MV: mechanical ventilation; SAPSII score = Simplified Acute Physiology Score within the first 48h after ICU admission;

^†^ GLASGOW score = Glasgow coma scale within the first 48h after ICU admission—scored even in patients receiving sedation—represents the level of awakening of patients; MDRPA = multi-drug-resistant *Pseudomonas aeruginosa*; MRSA = Methicillin-resistant *Staphylococcus aureus*; ESBLE: extended-spectrum βlactamase-producing *Enterobacteriaceae*

(†) p<0.001 for comparisons between the 3 categories of Glasgow score.

Factors that may lead to a decreased bronchial clearance or aspiration such as early enteral feeding or presence of a chest tube were only associated with EOP. On the contrary, severe organ dysfunction (low PaO2:FiO2 ratio) was associated with LOP.

Most antibacterial agents used within the first 48 hours of MV protected against EOP.The early administration of Vancomycin and imidazole, and fluoroquinolones administered between day 3 and 6 protected from LOP.

In term of previous carriage or colonization with multi-drug resistant pathogens, colonization with MDRPA was a risk factor for EOP; whereas colonization with MRSA was a risk factor for LOP. When MRSA colonization was present on admission, it had a lower impact than MRSA colonization was acquired later on, between the 3rd and 6th day of mechanical ventilation (15/34 vs 38/179, p<0.01).

Varying MV cut-off to five days for the definition of EOP and LOP had no impact on the final model (S2 Table).

## Discussion

Using commonly recognized definitions and quantitative bacterial samples, this study included a large multicenter population at risk for VAP, and aimed at identifying and differentiating risk factors associated with EOP and LOP, respectively. As one strategy for VAP prevention might be to decrease the involved risk factors, identifying the risk factors involved in VAP onset in each patient is critical. We used competing risk models in order to take account of the informative censor due to extubation for more than 48 hours and death. Among the mechanically-ventilated ICU patients included in our study, the incidence rate of VAP was about 19%, similar to the values reported in the literature [5, 10]. In patients with VAP, we observed rates of EOP and LOP of 40.2% and 59.8%, respectively, consistent with those observed in other studies [2, 18–20]. The distribution between EOP and LOP depends on the diagnostic method for pneumonia, and on the cut-off points used for the definition. We used a cut-off point of ≥ 7 days of MV, and the diagnosis of pneumonia was confirmed by either proximal or distal techniques with quantitative cultures of collected specimens. Varying the MV cut-off from seven to five days for the definition of EOP and LOP keeps sHR roughly unchanged in our study (S2 Table).

We observed a decrease in EOP but not in LOP over the three study time periods, concomitantly with an increase in the compliance with bundles of prevention measures in the participating centers. It might be a chronological coincidence; however, it is in line with a previous study that suggested that a bundle of preventive measures was more effective when implemented early after MV initiation[6].

Differences in the pathogenesis of each VAP types contribute to explain the differences in responsible pathogens. In the EOP, the lung is thought to be invaded by endogenous flora resulting from direct inoculation during emergency intubation or endotracheal tube manipulation, whereas pathogens involved in LOP originate from flora modified by hospitalization and antimicrobial treatment [5, 21]. The pattern of resistance highly depends on the level of resistance of the country or hospital. In countries with high rate of MDR pathogens, the difference between rates of MDR pathogens in LOP and EOP was not significant [22, 23]. Colonization with MDR bacteria represents a risk factor for the development of VAP [24]. In our study, colonization with MDR *P*. *aeruginosa* within the first 48 hours of MV was associated with a twice higher occurrence of EOP compared to that observed in non-colonized patients. It may reflect the fact that antibiotic used in the first days of mechanical ventilation were most often not active against MDR *P*. *aeruginosa*, and may induce colonization pressure. The colonization with MRSA, especially when it was acquired between the 3^rd^ and 6^th^ day of mechanical ventilation, was an important risk factor of LOP, with an incidence increased by 50% [21, 25]. Other studies demonstrated that nasal colonization with *S*. *aureus*, including MRSA, is a risk factor for the development of VAP [18]. The mechanisms by which previous colonization increased the risk of LOP is partly related to the protective role of previous antimicrobial therapy active on other microorganisms than MRSA. It could also be related to the decrease of prevention and of control of VAP, already shown to be associated with isolation [26]. The impact of the quantitative importance of colonization and the location of colonization was not available in our database and would require further studies.

Previous antimicrobial therapy was a protective factor of EOP but had marginal impact on the risk of LOP. The administration of betalactams, aminoglycosides or fluoroquinolones within the first 48 hours of MV protected from EOP. The maximal effect was obtained with carbapenems. Early administration of vancomycin protected from LOP, as did the use of fluoroquinolones between the 3^rd^ and the 6^th^ day of MV. This finding is consistent with previous studies results [11, 27]. It was shown that selective digestive decontamination with topical antibiotics combined with IV antibiotics during the first days of MV also decreased VAP rate [28]. In our study, the protective effect in the EOP risk of early anti-microbial against Gram-negative bacteria is counter-balanced by the deleterious impact of MDRPA carriage within the first 48 hours of mechanical ventilation.

In ICU patients with nosocomial pneumonia, compared to ICU patients without nosocomial pneumonia, a study identified local markers of lung immunosuppression, such as prolonged low expression of HLA-DR on alveolar macrophages and low cytokine levels [29]. Authors hypothesized that the development of nosocomial pneumonia might be associated with local organ immunosuppression. This post-aggressive general and pulmonary immunoparalysis may explain why both vasopressor use and low PaO2:Fi02 ratio remained important risk factors of LOP. Coma as the diagnosis for ICU admission was not a risk factor of EOP. However, the level of consciousness assessed in our study by the level of the effective Glasgow coma scale was associated with an increased risk of EOP. Our results suggest the need for awakening patients as early as possible during the ICU stay.

The use of enteral feeding in the first 48 hours of MV was a risk factor of EOP, and had no influence on LOP, therefore confirming previous work performed on patients with shock [30]. Early enteral nutrition may favor gastric regurgitation and inhalation of gastric content in case of intolerance, which may explain this result. Stress ulcer prophylaxis is associated with microbial colonization of the gastric juice and modification of the bacterial flora of the upper respiratory tract. Our study also suggests the lack of impact of proton pump inhibitor on the risk of VAP, consistently with a recent systematic review and meta-analysis [31]. The adverse impact of intra-hospital transport on risk of LOP is consistent with previous findings [32, 33]. The decrease of availability of tracheal suction, inadequate transport ventilator or pre-transport underestimation of patient' severity of illness may lead to more frequent occurrence of atelectasis and ventilator-associated pneumonia.

Our study had some limitations. VAP diagnosis in patients is difficult, as the standard for the diagnosis of VAP remains the histological examination and culture of lung tissue. We used bronchoscopic techniques or quantitative culture of endotracheal aspirates for bacteriological confirmation of the pneumonia diagnosis. We may have had a lower VAP rate compared to studies based only on clinical evaluations; however, this should impact equally EOP and LOP. The significant decrease of EOP over the study time periods is likely related to changes in prevention measures used in the study centers, but could also be due to unmeasured confounders, since we did not monitor the individual compliance with these measures. In addition, patients with a higher SAPSII score (>60) have a non-significant protection (sHR = 0.87), and the likely explanation is that they may die before any VAP onset. Finally, the impact of MDR and antimicrobial therapy refers to MDR risk observed in France, where carbapenemase-resistant *Enterobacteriaceae* strains are rare.

The major study strength is the use of the large, multicentric OUTCOMEREA database. This database contains patient's admission information and behavior during their ICUs stay, and comprises ICUs from university and non-university hospitals. Centers use a consistent VAP definition, which facilitates the generalization of our findings. In addition, the first episode of VAP was identified using the Fine and Gray model. This model allows a simultaneous estimation of two independent competing events: being extubated for more than 48 hours, death before occurrence of VAP.

## Conclusion

Despite the limitations discussed above, our study showed important findings, particularly the impact of colonization on VAP occurrence and the protective effects of antibiotics administered during the first 48 hours of MV. Factors associated with a quantitative increase of aspiration of the oropharyngeal content were predominant for EOP. In LOP the persistence of organ system failures and shock, and colonization with MRSA were the main risk factors. VAP risk factors are mostly different according to the pneumonia time of onset, which should lead to differentiated prevention strategies.

## Supporting information

S1 File (CSV)

S2 File (CSV)

S1 Table Summary of procedures used for ventilator associated pneumonia prevention in the ICUs of the OUTCOMEREA network. (DOCX)

S2 Table Risk factors of early pneumonia using a cut-off at 5 days of mechanical ventilation. (DOCX)
