# Supplementary material for: Respective impact of implementation of prevention strategies, colonization with multiresistant bacteria and antimicrobial use on the risk of early- and late-onset VAP: An analysis of the OUTCOMEREA network
Source: PLoS One. 2017 Nov 29;12(11):e0187791. doi: 10.1371/journal.pone.0187791 (PMC5706682; doi:10.1371/journal.pone.0187791)
Supplement: S1 Table — (DOCX) [file pone.0187791.s003.docx]

**S1 Table: Summary of the procedure used for Ventilator associated pneumonia prevention ICUs of the OUTCOMEREA network.**

|  | ***Before 2001*** | ***2001-2006*** | ***After 2006*** |
| --- | --- | --- | --- |
| Daily weaning trials | 13% | 50% | 75% |
| Daily discontinuation of narcotic and analgesia if no contra-indications | 13% | 25% | 25% |
| Nurse driven protocolized sedation | 13% | 25% | 25% |
| Early tracheostomy | 13% | 13% | 0% |
| Orogastric tube | 50% | 75% | 88% |
| Oral care with chlorhexidine | 14% | 29% | 57% |
| Early enteral nutrition | 54% | 71% | 100% |
| Protun pump inhibitor use for gastric protection | 14% | 57% | 100% |
| Minimal level of peep> 5 mmHg | 50% | 88% | 100% |
| Selective digestive decontamination | 0% | 0% | 0% |
| Selective oral decontamination | 0% | 0% | 0% |
| Closed suction systems | 13% | 38% | 50% |
| Protocolized ventilation during intra-hospital transports | 13% | 38% | 75% |

Investigators from the Outcomerea database were questioned about the elements of prevention bundles that were applied in their ICU at different time period. The compliance with the prevention strategy was not systematically checked.
