# Supplementary material for: Respective impact of implementation of prevention strategies, colonization with multiresistant bacteria and antimicrobial use on the risk of early- and late-onset VAP: An analysis of the OUTCOMEREA network
Source: PLoS One. 2017 Nov 29;12(11):e0187791. doi: 10.1371/journal.pone.0187791 (PMC5706682; doi:10.1371/journal.pone.0187791)
Supplement: S2 Table — (DOCX) [file pone.0187791.s004.docx]

S2 Table: Risk factors of early pneumonia using a cut-off at 5 days of mechanical ventilation

| Characteristics | **sHR** | **P** |
| --- | --- | --- |
| Male gender | 1.23[0.95-1.59] | 0.1170 |
| Renal chronic diseases at ICU admission | 1.40[0.86-2.26] | 0.1773 |
| SAPSII score ( ref: ≤38) |  |  |
| 37-48 | 1.06[0.76-1.47] | 0.7304 |
| 49-59 | 1.09[0.78-1.54] | 0.6116 |
| ≥60 | 0.89[0.62-1.29] | 0.5428 |
| Effective Glasgow†(ref>13) |  |  |
| ≤ 5 | 1.43[0.90-2.27] | 0.1266 |
| 5>Glasgow<13 | 1.34[0.84-2.14] | 0.2134 |
| Period effect (ref: <2001) |  |  |
| 2001-2006 | 0.99[0.63-1.58] | 0.9836 |
| ≥ 2007 | 0.74[0.46-1.18] | 0.2086 |
| Variables within the first 48 hours of MV | | |
| MDR PA Microbial colonization | 1.59[0.60-4.18] | 0.3483 |
| Treatments and procedures |  |  |
| Chest tube | 1.14[0.74-1.75] | 0.5516 |
| Enteral feeding | 1.45[1.11-1.91] | 0.0075 |
| Antibacterial agents administration |  |  |
| 3^rd^ and 4^th^ generation cephalosporins | 0.356[0.26-0.52] | <.0001 |
| ß-lactam/ ß-lactamase inhibitor | 0.50[0.37-0.67] | <.0001 |
| Other penicillins | 0.53[0.32-0.88] | 0.0133 |
| Penems | 0.17[0.06-0.47] | 0.0005 |
| Fluoroquinolones | 0.60[0.39-0.92] | 0.0193 |
| Aminoglycosides | 0.49[0.32-0.75] | 0.0012 |
| Vancomycin | 0.77[0.47-1.26] | 0.2987 |

SAPSII score= Simplified Acute Physiology Score within the first 48h after ICU admission; † GLASGOW score = Glasgow coma scale within the first 48h after ICU admission - scored even in patients receiving sedation - represents the level of awakening of patients ; MDRPA = multi-drug-resistant *Pseudomonas aeruginosa*
